# IRF7 Regulates TLR2-Mediated Activation of Splenic CD11c^hi^ Dendritic Cells

**DOI:** 10.1371/journal.pone.0041050

**Published:** 2012-07-16

**Authors:** Benjamin M. J. Owens, John W. J. Moore, Paul M. Kaye

**Affiliations:** Centre for Immunology and Infection, Hull York Medical School and Department of Biology, University of York, York, United Kingdom; Oklahoma Medical Research Foundation, United States of America

## Abstract

Members of the Interferon Regulatory Factor (IRF) family of transcription factors play an essential role in the development and function of the immune system. Here we investigated the role of IRF7 in the functional activation of conventional CD11c^hi^ splenic dendritic cells (cDCs) *in vitro* and *in vivo*. Using mice deficient in IRF7, we found that this transcription factor was dispensable for the *in vivo* development of cDC subsets in the spleen. However, IRF7-deficient cDCs showed enhanced activation in response to microbial stimuli, characterised by exaggerated expression of CD80, CD86 and MHCII upon TLR2 ligation *in vitro*. The hyper-responsiveness of *Irf7* ^−/−^ cDC to TLR ligation could not be reversed with exogenous IFNα, nor by co-culture with wild-type cDCs, suggesting an intrinsic defect due to IRF7-deficiency. *Irf7* ^−/−^ cDCs also had impaired capacity to produce IL-12p70 when stimulated *ex vivo*, instead producing elevated levels of IL-10 that impaired their capacity to drive Th1 responses. Finally, analysis of bone marrow microchimeric mice revealed that cDCs deficient in IRF7 were also hyper-responsive to TLR2-mediated activation *in vivo*. Our data suggest a previously unknown function for IRF7 as a component of the regulatory network associated with cDC activation and adds to the wide variety of situations in which these transcription factors play a role.

## Introduction

Interferon regulatory factor (IRF) 7, a member of the IRF family of transcription factors, is the ‘master regulator’ of type I interferon (IFN) - dependent immune responses and underpins their critical role in host defence [1]. However, IRFs are also essential for the full development of many components of the immune system [2], including dendritic cells (DCs). DCs are critical for the initiation and control of adaptive immunity [3], responding to infectious insult by changes in expression of MHCII, costimulatory molecules (notably of the B7 family) and cytokines involved in directing CD4^+^ and CD8^+^ T cell differentiation [4], [5]. In the murine spleen, CD11c^hi^ ‘conventional’ DCs (cDCs) can be segregated into three distinct subsets based upon surface expression of CD4 and CD8α [6], [7]. Several members of the IRF family are known to be critical for the faithful development of these murine cDC subsets *in vivo*. Splenic CD8α^+^ cDC development is exquisitely dependent on the expression of IRF8 [8], [9] whereas differentiation of splenic CD4^+^ cDCs depends on IRF4 [10], [11]. DN cDCs express and at least partially rely on both IRF4 and IRF8 for their full development [10]. Non-lymphoid DCs such as Langerhans cells and dermal DCs also require IRF8 expression for normal development *in vivo* [12]. In addition, *Irf2^−/−^* mice selectively lack splenic CD4^+^ cDCs and epidermal DC subsets [13], whereas IRF1-deficient animals have a reduced number of splenic CD8a^+^ cDCs [14].

Although Type I IFNs are known to augment costimulatory molecule expression by DCs and monocytes in mice and humans [15], [16], [17], [18], the specific IRFs involved are less clearly defined. PD-L1 and CD40 expression on endothelial cells is critically dependent on IRF1 [19], [20], with this transcription factor also required for CD80 expression by monocytes *in vitro* – a situation where IRF7 appears to be redundant [21]. Nevertheless, there is some evidence supporting an interaction between IRF7 and costimulatory molecules, with an IRF7 binding site identified in the CD80 promoter that is involved in regulation of CD80 expression in LPS stimulated human monocytes [22]. Furthermore, large scale analysis of genes regulated by IRF7 in response to viral infection have identified CD80 as a potential target of this transcription factor in an *in vitro* system, suggesting a link between IRF7 and expression of certain costimulatory molecules [23]. However, whether IRF7 regulates the activation of splenic cDCs, and in particular their expression of costimulatory molecules, is currently unknown.

Here, we first sought to determine whether this transcription factor was required for the development of cDC subsets in the murine spleen. Furthermore, in light of the proposed link between IRF7 and the regulation of CD80 expression and possible modulation of Toll-like receptor (TLR) signalling by type I IFNs [24], we next sought to elucidate the role of this transcription factor in the functional activation of splenic cDCs in response to TLR ligation. We show that IRF7-deficient cDCs are hyper-responsive to TLR2, responding with heightened CD86 expression in* vitro* and *in vivo*, in addition to altered cytokine profiles that impact on their CD4^+^ T cell polarising capacity. These data implicate IRF7 as a component of a previously unknown regulatory pathway initiated in DCs during their response to microbial stimuli.

## Results

### Faithful Splenic cDC Development and Equivalent Steady-state TLR2 Expression in the Absence of IRF7

**Figure 1:**
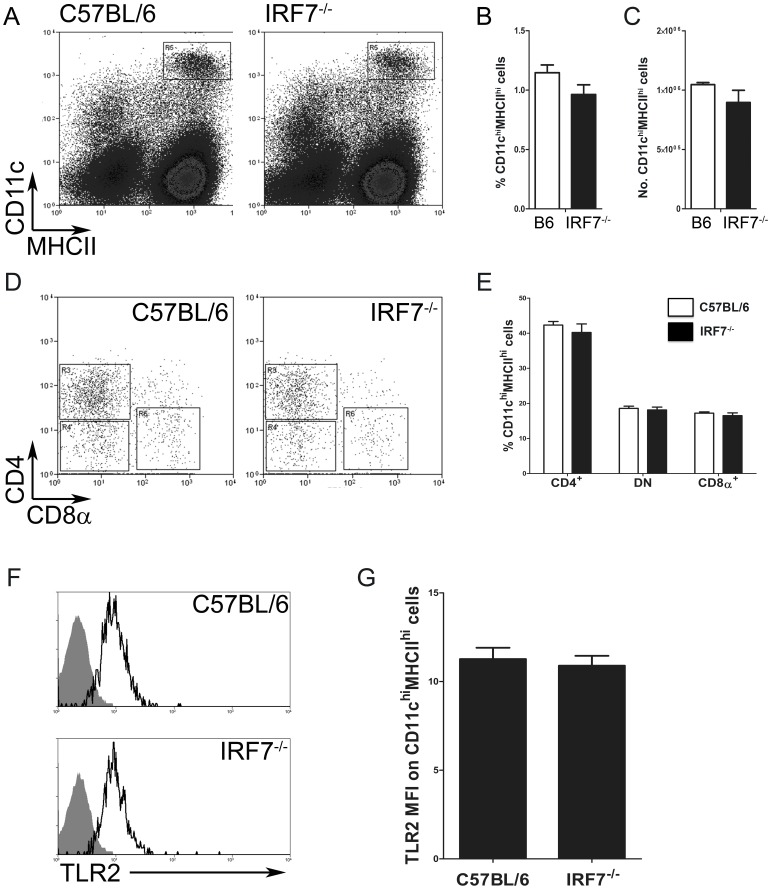
Splenic cDC subset development and TLR2 expression is independent of IRF7. The presence of splenic CD11c^hi^MHCII^hi^ cDCs in C57BL/6 and B6.*Irf7* ^−/−^ mice was determined by flow cytometry (**A** & **B**). CD4^+^, DN and CD8α^+^ cDC subset frequency was determined in the CD11c^hi^MHCII^hi^ compartment of wildtype and IRF7-deficient animals (**C** & **D**). Expression of TLR2 on CD11c^hi^MHCII^hi^ cells from C57BL/6 and B6.*Irf7* ^−/−^ mice was determined by flow cytometry (**E** & **F**). Flow plots and histograms in **A**, **C** and **E** are representative, data in **B**, **D** and **F** show mean frequency ± SEM of indicated cell population in C57BL/6 (open bars) and B6.*Irf7* ^−/−^ (closed bars) mice. Histograms in **E** are gated on CD11c^hi^MHCII^hi^ cells gated as in **A** and show specific TLR2 staining (open line) compared to isotype control antibody (filled histogram). Data are from n = 4–5 mice per group and representative of three separate experiments.

Mice deficient in IRF7 had a normal complement of hematopoietic cells in the liver [25], normal splenic architecture [26] and had no major alterations in the frequencies of CD4^+^ and CD8^+^ T cells, CD19^+^ B cells, CD11b^+^ macrophages, Gr-1^+^ neutrophils and DX5^+^NKp46^+^ NK cells, compared to wild type control mice (**Supplementary Fig. S1** and data not shown). Analysis of collagenase-digested spleens from C57BL/6 and B6.*Irf7^−/−^* mice revealed normal populations of CD11c^hi^MHCII^hi^ cDCs in both strains (**Fig. 1A**). cDCs were present in comparable frequencies (1.15±0.06% and 0.96±0.08% of total splenocytes in C57BL/6 and B6.*Irf7^−/−^* mice, respectively; **Fig. 1B**) and number (1.05×10^6^±1.72×10^4^ and 8.96×10^5^±1.02×10^5^ in C57BL/6 and B6.*Irf7^−/−^* mice respectively; **Fig. 1C**). All CD11c^hi^ cDCs subsets were present in the absence of IRF7 (**Fig. 1D**) and were at the expected frequencies [6], [7] (**Fig. 1E**). CD4^+^ cDCs comprised 42.32±1.01% of CD11c^hi^MHCII^hi^ cells in C57BL/6 and 40.20±2.43% in B6.*Irf7^−/−^* mice, respectively. CD4^-^CD8α^-^ (double negative, DN) cDCs were similarly unaffected by IRF7 deficiency, making up 18.57±0.62% of splenic cDCs in wild-type mice and 18.11±0.81% in those lacking IRF7. The CD8α^+^ subset developed normally in the absence of IRF7, comprising 17.19±0.38% of steady state splenic cDCs in C57BL/6 mice and 16.48±0.79% in B6.*Irf7^−/−^* mice. To determine whether IRF7 deficiency altered the capacity for TLR2 expression by cDCs, CD11c^hi^MHCII^hi^ cDCs from steady-state wildtype and B6.*Irf7* ^−/−^ mice were assessed for surface expression of TLR2. cDCs from both strains expressed equivalent amounts of TLR2 (**Fig. 1F and G**). Therefore, IRF7 is not a critical transcription factor for the development of splenic cDCs subsets *in vivo* and expression of TLR2 is not affected by its absence.

### IRF7 Deficiency Leads to Dendritic Cell Hyper-activation *in vitro*

**Figure 2:**
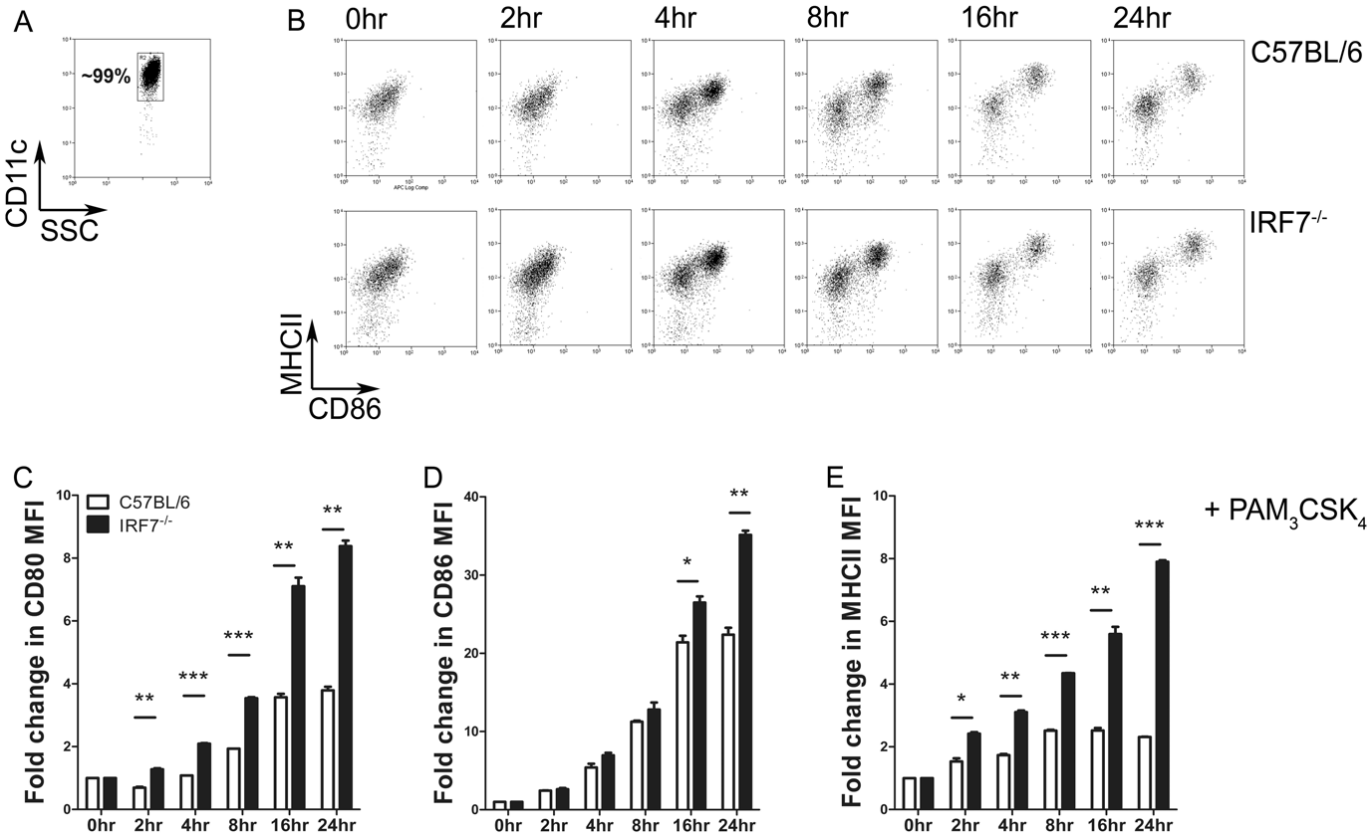
IRF7-deficient splenic cDCs are hyper-responsive to a TLR2 agonist *in vitro*. A . CD11c^hi^ cDCs were sorted to **∼**99% purity from the spleens of C57BL/6 and B6.*Irf7* ^−/−^ mice. Cells were cultured in triplicate at 1×10^6^ cells/ml in the presence of 10 µg/ml PAM_3_CSK_4_. At the indicated times post-stimulation, cells were removed and assessed by flow cytometry for expression of CD80, CD86 and MHCII. Representative flow plots showing progressive cDC activation in terms of CD86 and MHCII expression are shown in **B**, fold increases in surface expression of CD80, CD86 and MHCII on cDCs at the indicated time point over unstimulated cDCs are shown in **C**, **D** and **E**, respectively. **C**–**E** show mean fold increase ± SEM in surface expression of indicated proteins on cDCs from C57BL/6 (open bars) or B6.*Irf7* ^−/−^(closed bars) mice, compared to unstimulated cDCs from the same strain. Data are pooled from three individual experiments. * = p<0.05 ** =  p<0.01, *** = p<0.001.

We next sought to determine the impact of a lack of IRF7 on the activation state of splenic cDCs in response to TLR2 ligation *in vitro*. We sorted CD11c^hi^ cDCs to >98% purity from the spleens of C57BL/6 and B6.*Irf7^−/−^* mice and cultured them for a total of 24 hrs in the presence of the TLR2/6 agonist PAM_3_CSK_4_ (**Fig. 2A**). Cells were monitored at intervals by flow cytometry for activation, as measured by fold changes in surface expression of MHCII, CD80 and CD86 (**Fig. 2B**). Stimulation of cDCs from both strains led to their activation, as indicated by progressively increasing expression of all three surface markers. However in all cases, cDCs which lacked expression of IRF7 were hyper-activated in response to TLR2 stimulation, with significantly greater fold increases in expression of CD80 (**Fig. 2C**), CD86 (**Fig. 2D**) and MHCII (**Fig. 2E**) when compared with IRF7-sufficient cDCs stimulated in the same way. Such hyper-activation was not consistently observed after stimulation with agonists for TLR3, TLR4 or TLR9 (**Supplementary Fig. S2**). Hyper-activation in response to TLR2 triggering was evident from 2h post treatment in the case of CD80 and MHCII expression, when assessed on the bulk population. In all experiments there was some heterogeneity in cDC upregulation of co-stimulatory molecules in response to TLR triggering, however enhanced MHCII and CD86 expression by B6.*Irf7* ^−/−^ cDCs was also apparent when gating only on the more activated population, and the proportion of activated cDCs within the whole population was consistently higher in the IRF7-deficient groups at all time points after stimulation (**Supplementary Fig. S3**). By 24 h post activation, the increase in expression of these markers of activation on cDCs from B6.*Irf7* ^−/−^ (as measured by changes in MFI on the whole population) was **∼**2-fold (CD80) and **∼**4-fold (MHCII) greater than seen with cDC isolated from C57BL/6 mice. For example, whereas TLR2 activated cDCs from C57BL/6 mice increased expression of CD80 by 3.79±0.12 fold compared to resting levels by 24 h, cDCs isolated from B6.*Irf7* ^−/−^ mice increased expression of CD80 by 8.38±0.18 fold after the same stimulus (p<0.01). In the case of CD86 expression, an impact of IRF7-deficiency was only evident at later time points, and was most pronounced when considering the more activated of the cDC subpopulations (**Fig. 2B and D**, and **Supplementary Fig. S3D**). Collectively, these data indicate that in the absence of IRF7, splenic cDCs become hyper-activated in response to TLR2 stimulation *in vitro*, showing significantly exaggerated expression of the costimulatory molecules CD80 and CD86, as well as of MHCII.

### Exaggerated CD86 Expression by *Irf7* ^−/−^ cDCs Occurs in the Presence of Wildtype cDCs and Exogenous IFNα

**Figure 3:**
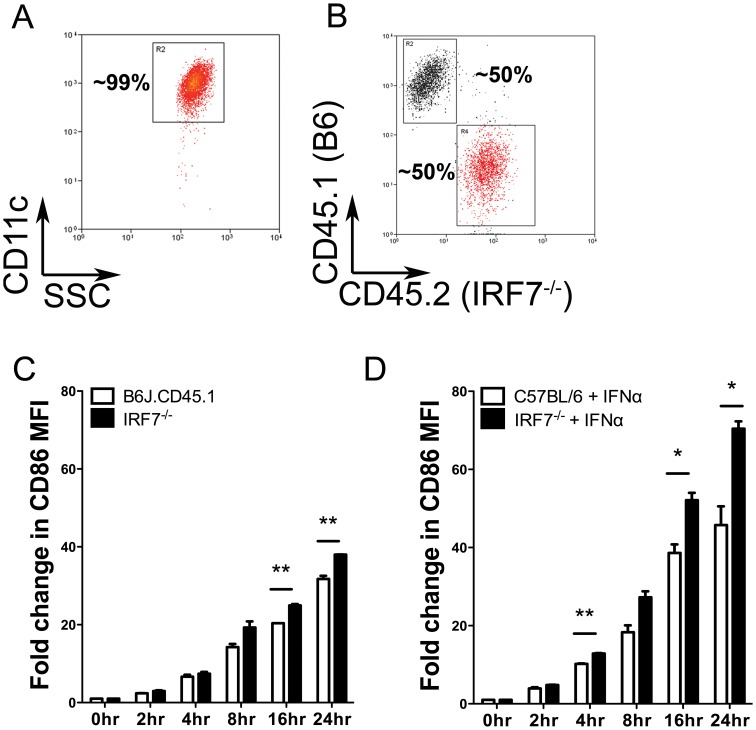
Exaggerated CD86 expression after TLR2 stimulation occurs in the presence of IRF7-sufficient cDCs or exogenous IFNα. A. Representative purity of splenic CD11c^hi^ cDCs sorted from C57BL/6, B6.*Irf7* ^−/−^ and congenic B6J.CD45.1 mice. B. Representative staining of cDCs from B6.*Irf7* ^−/−^ and B6J.CD45.1 mice after co-culture at **∼**50∶50 ratio in the presence of 10 µg/ml PAM_3_CSK_4_. C. The fold increase in expression of CD86 after TLR2 stimulation for cDCs from either strain was determined by flow cytometry at the indicated times post-stimulation. D. C57BL/6 or B6.*Irf7* ^−/−^ cDCs were cultured in the presence of 10 µg/ml PAM_3_CSK_4_ and 1000 U/ml IFNα. At the indicated times post- stimulation, cells were removed and assessed by flow cytometry expression of CD86. Data are presented as mean fold increase ± SEM in surface expression of CD86 relative to unstimulated cDCs from the same strain. Data are from two experiments. * = p<0.05 ** =  p<0.01, *** = p<0.001.

IRF7 regulates the production of Type I IFNs and potentially other soluble factors that could normally act in a negative feedback loop to self-limit activation. We tested this hypothesis in two ways. First, we first sorted CD11c^hi^ cDCs from B6J.CD45.1 and congenic (CD45.2) B6.*Irf7* ^−/−^ mice (**Fig. 3A**). Sorted cDCs were then co-cultured at an approximately 50∶50 ratio (**Fig. 3B**) and stimulated with PAM_3_CSK_4_, as before. Gating on CD45.1 or CD45.2 during flow cytometric analysis allowed an assessment of activation (measured as the fold increase in co-stimulatory molecule expression comparing stimulated to unstimulated cells) on wildtype and IRF7-deficient cDCs cultured in the same microenvironment. Even in the presence of wildtype cells, cDCs from IRF7-deficient mice remained hyper-responsive to TLR2 activation, as assessed for CD80, CD86 and MHCII expression (**Fig. 3C** **and data not shown**). Next, we directly examined the potential role of Type I IFN as a negative regulator of CD86 expression. Addition of a biologically active amount of exogenous IFNα [26] potentiated the response to TLR2 ligation in wildtype cDC, inducing an increase in surface expression approximately twice that observed by TLR2 stimulation alone (**Fig. 3C and D**). Importantly, IRF7-deficient cDCs retained their exaggerated response to TLR2 stimulation even in the presence of exogenous IFNα, as measured by CD86 (**Fig. 2D** **),** as well as CD80 and MHCII expression (data not shown). Therefore, addition of neither wildtype cDCs nor exogenous IFNα was able to prevent the hyper-activation of B6.*Irf7* ^−/−^ cDCs in response to TLR2 stimulation, suggesting that IRF7 may act as a cell intrinsic endogenous regulator of TLR-mediated activation in splenic cDCs.

### IRF7 Modulates TLR-mediated Cytokine Production by Splenic cDCs *ex vivo*

**Figure 4:**
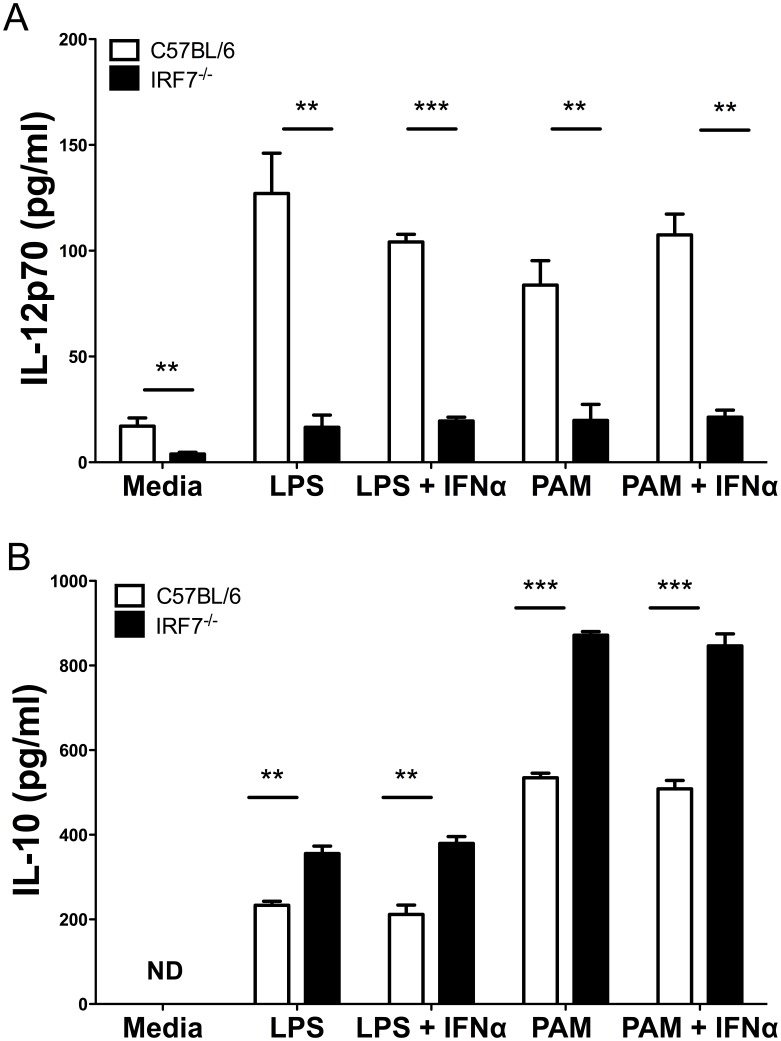
Cytokine production by IRF7-deficient cDCs *in vitro*. CD11c^hi^ cDCs were sorted from spleens of C57BL/6 and B6.*Irf7* ^−/−^ mice and cultured for 24 hours in the presence of 10 µg/ml PAM_3_CSK_4_, 1 µg/ml LPS and 1000U/ml IFNα, or combinations thereof. Supernatants were assessed by ELISA for presence of IL-12p70 (**A**&**C**) or IL-10 (**B**&**D**). Data show mean concentration of cytokine from triplicate wells ± SEM and are representative of two experiments. ** = p<0.01 *** = p<0.001.

To extend this functional assessment of the role of IRF7 in cDC activation, CD11c^hi^MHCII^hi^ splenic cDCs were sorted from naïve C57BL/6 and B6.*Irf7^−/−^* mice and cultured for 24 hours in the presence of LPS or PAM_3_CSK_4_ with or without exogenous IFNα (**Fig. 4**). Levels of IL-12p70 (**Fig. 4A**) and IL-10 (**Fig. 4B**) were determined in culture supernatant by ELISA. IL-12p70 production by cDCs due to spontaneous maturation in culture was impaired when cDCs lacked IRF7 (17.1±3.86 pg/ml vs. 3.94±0.82 pg/ml in C57BL/6 and B6.*Irf7* ^−/−^ cDCs respectively; p<0.01). In response to stimulation with LPS, IRF7-deficiency severely impaired IL-12p70 production by cDCs (127.06±19.08 pg/ml vs. 16.51±5.85 pg/ml after 24 h culture; p<0.01). The addition of exogenous IFNα affected neither the response of wild type cDCs to LPS stimulation nor the defective response of B6.*Irf7* ^−/−^ cDCs (**Fig. 4A**). Similarly impaired IL-12p70 production was also observed in IRF7-deficient cells cultured with PAM_3_CSK_4_ and again exogenous IFNα did not compensate for this deficiency. In contrast to the defective IL-12p70 production observed in IRF7-deficient cDCs, culture supernatants of cDCs isolated from B6.*Irf7^−/−^* mice contained significantly higher levels of IL-10 when stimulated with either LPS or PAM_3_CSK_4_ (**Fig. 4B**). As with TLR induced IL-12p70, we found no evidence to suggest that exogenous IFNα affected production of IL-10 in either strain of mice. Therefore, a lack of IRF7 in splenic cDCs leads to reciprocal effects on two key cytokines involved in T cell activation and differentiation and produced by cDCs in response to TLR2 and TLR4 ligands *in vitro*.

### IL-10-dependent Impairment in Th1 Polarisation by IRF7-deficient cDCs *in vitro*

**Figure 5:**
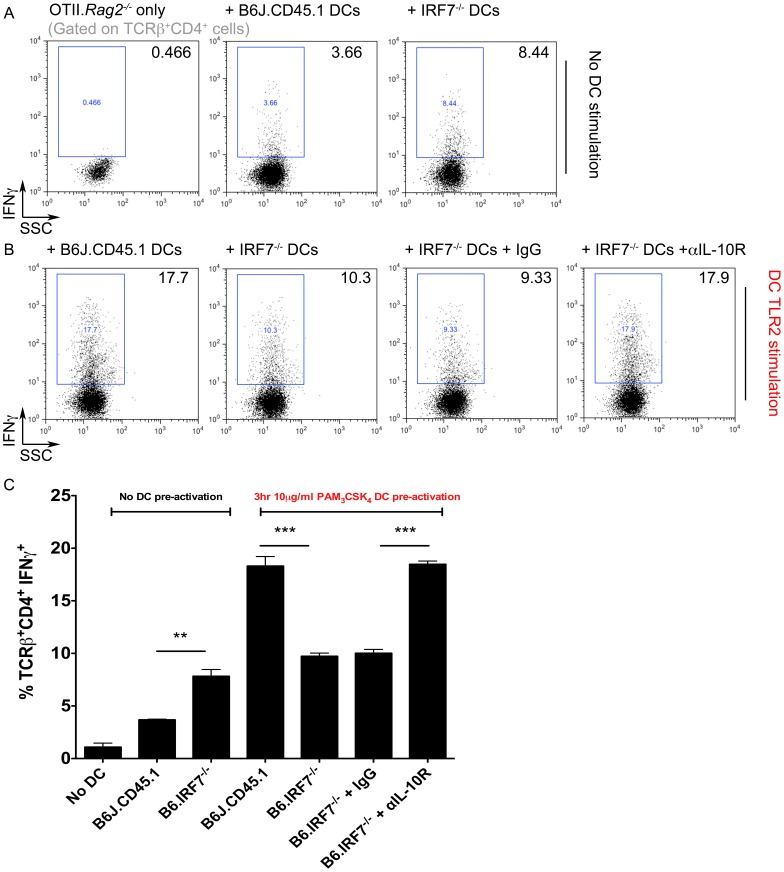
IL-10 dependent impairment in Th1 polarisation by IRF7-deficient cDCs. Splenic CD11c^hi^ cDCs were purified from C57BL/6 and B6.*Irf7* ^−/−^ mice and cultured for 3 hours in the presence/absence of 10 µg/ml PAM_3_CSK_4_. DCs were cultured at a 1∶2 ratio with CFSE-labeled CD4^+^ OTII.*Rag2* ^−/−^ cells for 7 days. TCRβ^+^CD4^+^ T cells were assessed for production of IFNγ after *in vitro* restimulation from cultures containing unstimulated DCs (**A**&**C**) or DCs pre-activated for 3 hours with PAM_3_CSK_4_ (**B**&**C**). Dot plots in **A** and **B** are representative, **C** shows mean percentage of IFNγ^+^ OTII.*Rag2^−/−^* T cells in indicated culture conditions ± SEM. Each condition contained 3 replicate cultures, using DCs purified and pooled from 8–9 individual mice of each genotype. ** = p<0.01 *** = p<0.001.

To determine the functional significance of the altered activation state and cytokine profile of IRF7-deficient cDCs after TLR2 triggering, we employed a TCR transgenic co-culture approach (**Fig. 5**). CD11c^+^ cells were enriched from spleens of C57BL/6 or B6.*Irf7* ^−/−^ mice, stimulated (or not) with PAM_3_CSK_4_ and cultured with CFSE-labelled CD4^+^ OTII.*Rag2* ^−/−^ splenic T cells. After 7 days, cultures were restimulated and OTII T cells assessed for proliferation and cytokine production by flow cytometry. IRF7-deficient cDCs stimulated significantly more proliferation in OTII cells than cDC from wild-type mice, likely reflecting a degree of spontaneous activation during culture that was regulate by IRF7, but this difference was lost after *in vitro* TLR2 stimulation, (data not shown). Similarly, resting B6.*Irf7* ^−/−^ cDCs had an enhanced capacity to drive OTII T cells toward a Th1 phenotype compared to wild-type cells (**Fig. 5A&C**). However, after cDCs were stimulated with PAM_3_CSK_4_, cells lacking IRF7 exhibited a significantly impaired capacity to drive Th1 polarisation and IFNγ production by OTII T cells, when compared to those from wild-type mice (p<0.001, **Fig. 5B&C**).

To assess the impact of the dysregulated cytokine expression by B6.*Irf7* ^−/−^ cDCs after TLR2 triggering (Fig. 4) on Th1 polarisation, we modulated cytokine levels in the co-cultures (**Fig. 5B&C**). Addition of recombinant IL-12 was unable to rescue the defect in Th1 polarisation by B6.*Irf7* ^−/−^ cDCs (data not shown). However, blocking IL-10 signalling by including an αIL-10R mAb allowed B6.*Irf7* ^−/−^ cDCs to polarise CD4^+^ T cells toward an IFNγ^+^ phenotype at an equivalent efficiency to wild-type cDCs. Thus unstimulated B6.*Irf7* ^−/−^ cDCs have a slightly enhanced capacity to activate CD4^+^ T cells *in vitro,* and after TLR2 triggering, B6.*Irf7* ^−/−^ cDCs exhibit an IL-10-dependent impairment in Th1 polarising capacity.

### *In vivo* Administration of PAM_3_CSK_4_ to Microchimeric Mice Reveals Enhanced CD86 Expression on IRF7-deficient cDCs

**Figure 6:**
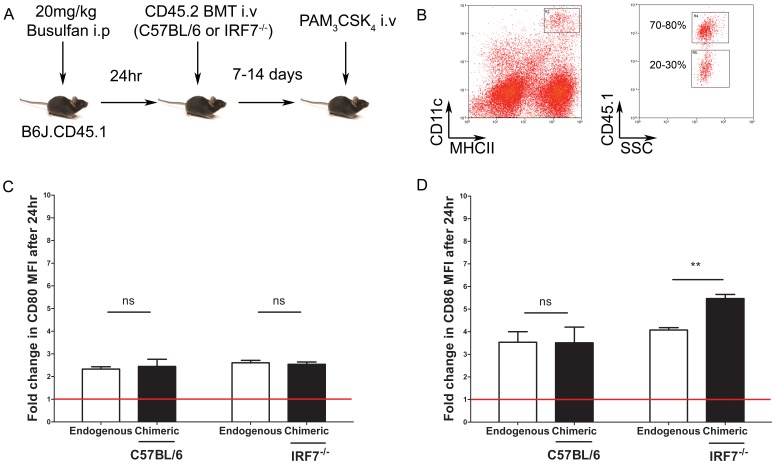
IRF7-deficient cDCs show hyper-responsiveness to PAM_3_CSK_4_ *in vivo* even in an IRF7-sufficient environment. B6.*Irf7* ^−/−^ or C57BL/6 microchimeric mice were generated by bone marrow transfer into Busulfan-treated congenic B6J.CD45.1 hosts. **A**. 7 to 14 days post- engraftment, microchimeric mice bearing B6.*Irf7* ^−/−^ or C57BL/6 chimeric cell populations received 5 µg/mouse PAM_3_CSK_4_ intravenously. Splenic CD11c^hi^MHCII^hi^ cDC compartments in microchimeric animals were comprised of endogenous (CD45.1^+^) and chimeric (CD45.1^-^) cell populations (**B**). After 24 hours, activation of splenic cDCs after PAM_3_CSK_4_ administration *in vivo* was assessed by quantifying changes in surface expression of CD80 (**C**) and CD86 (**D**) by flow cytometry. Flow plots in **B** are representative. Data in **C** and **D** show the mean fold change in indicated surface protein ± SEM on endogenous (open bars) and chimeric (black bars) cDCs after PAM_3_CSK_4_ injection, compared to the same populations of cDCs in mice receiving PBS. n = 4 per group, representative of two experiments. ** =  p<0.01.

We next sought to address the role of IRF7 in regulating cDC activation by TLR2 *in vivo*. To investigate this, we generated microchimeric mice in which a minor population of donor derived hematopoietic cells develop under physiological conditions. B6J.CD45.1 mice were injected i.p. with the stem cell depleting drug busulfan [27], [28], and the next day bone marrow cells from congenic CD45.2^+^ donor C57BL/6 or B6.*Irf7* ^−/−^ mice were adoptively transferred (**Fig 6A**). After 7–14 days to allow engraftment and the development of donor derived cDCs, microchimeric mice were injected with PBS or PAM_3_CSK_4_ i.v. and 24h later, cDCs of recipient (i.e. untouched endogenous cDCs) and donor origin were assessed for expression of CD80 and CD86 by flow cytometry. Spleens of microchimeric mice contained CD11c^hi^MHCII^hi^ cDCs derived from both host (CD45.1) and donor (CD45.2) genotypes (**Fig. 6B**). There was no difference in the ability of C57BL/6 or B6.*Irf7* ^−/−^ bone marrow to engraft, with equivalent frequencies of endogenous (**∼**70–80%) vs. chimeric (**∼**20–30%) cDCs regardless of which strain of donor mice was used (data not shown). For each population of cDCs, we calculated the fold change in expression for CD80 or CD86 in mice that had received PAM_3_CSK_4_ compared to those that had received saline alone injection. Although *in vivo* administration of PAM_3_CSK_4_ increased the expression of CD80 on the endogenous cDCs by approximately two fold compared to untreated mice, there was no significant difference in extent of upregulation of CD80 between wildtype and IRF7-deficient chimeric cDCs (**Fig. 6C**). As expected, in vivo administration of PAM_3_CSK_4_ -enhanced CD86 expression on the endogenous population of cDCs (by **∼**3.5 fold), and expression was als increased to the same extent on wild type chimeric cDCs. In contrast, chimeric IRF-7-deficient cDCs displayed a hyper-activated phenotype, with enhanced CD86 expression relative to the endogenous population of cDCs (**Fig. 6D**), of similar magnitude to that seen in vitro (**Fig. 2D**). Hence, cDCs lacking IRF7 can be hyper-activated either in vitro or in vivo (where they have developed and been activated in a fully IRF7-sufficient environment), but environment (i.e. *in vitro* or *in vivo*) determines the extent to which CD80 and CD86 are affected.

## Discussion

This study reveals a novel role for IRF7 in regulating TLR2-induced costimulatory molecule expression and cytokine production by splenic cDCs *in vitro* and *in vivo*. Unlike many other members of this transcription factor family, IRF7 is redundant with respect to the *in vivo* development of cDC subsets. However, cDCs lacking IRF7 respond to TLR2 activation with exaggerated costimulatory molecule expression and altered cytokine production *in vitro*, impacting on their capacity to affect CD4^+^ T cell polarisation. Furthermore, administration of PAM_3_CSK_4_ to microchimeric mice indicates a key role for IRF7 in the regulation of CD86 expression by splenic cDCs *in vivo*.

IRF7 appears to play a limited role in the development of cDCs subsets and other splenic immune cell types, supporting previous data showing normal cell distribution in the spleens of B6.*Irf7^−/−^* mice by immunohistochemistry [26] and in the livers of IRF7-deficient mice by flow cytometry [25]. This is in stark contrast to mice lacking expression of other IRF family members, with normal cDC, CD8α^+^ T cell [29], [30] and NK cell [31], [32], [33] development relying on the expression of several IRFs. These data suggest either that compensatory mechanisms for immune cell development exist in the absence of IRF7, or that the functions of this transcription factor are more restricted than other members of the same family.

As IRF7 is critical for the optimal induction of type I IFN expression, it was surprising that a deficiency in this transcription factor led to alterations in the response of cDCs to ligation of TLR2; signalling through which was, until recently, not thought to lead to type I IFN expression [34]. However it is now becoming clear that TLR2 signalling can lead to type I IFN production under some conditions. This has been observed during the response of mice to infection with vaccinia virus, where a subset of Ly6C^hi^ inflammatory monocytes produces type I IFN in an IRF3 and IRF7-dependent manner after TLR2-mediated recognition of viral ligands [35]. This was proposed to be restricted to both inflammatory monocytes and virus-derived stimuli. However, more recent studies have revealed a capacity for type I IFN production in other cell types and in response to diverse TLR2 ligands, including PAM_3_CSK_4_ [36], [37]. This is thought to be dependent upon the localisation of TLR2 to endolysosomal membranes, with subsequent signalling from this compartment leading to IFNα/β production - more akin to the mechanisms employed by TLR3, TLR7 and TLR9 [38], [39]. The location of TLR2 to endosomal compartments and subsequent IFNα/β production may, as with TLR4, be dependent upon TRAF3, as forced localisation of TRAF3 to the plasma membrane enables IFNβ production after PAM_3_CSK_4_ stimulation, rather than the classical plasma membrane-restricted signalling that initiates pro-inflammatory cytokine production in response to TLR2 [40]. However it is still unclear as to the precise molecular mechanisms leading to TLR2-induced IFNα/β production.

Although IFNα production is critically dependent upon IRF7, IFNβ production in response to LPS stimulation occurs at normal levels in IRF7-deficient cells [1]. However, the recent studies showing type I IFN production in response to endosomal TLR2 signalling all revealed a key requirement for IRF7 in the induction of IFNβ [36], [37], [41]. Therefore, in addition to a lack of IFNα, cDCs stimulated with TLR2 ligands would also likely lack the capacity to produce IFNβ. Contradictory findings have been reported regarding the role of type I IFNs in regulating CD80/86 expression. IFNβ has been shown to be required for maximal costimulatory molecule expression on peritoneal macrophages in response to LPS stimulation [16], whereas splenic cDCs which are deficient in the IFNαβ receptor (IFNαβR) and thus cannot respond to either type I IFN, show greatly enhanced expression of CD80 and CD86 in response to infection with *Listeria monocytogenes in vivo* [42]. The data presented here show that IRF7 deficiency led to exaggerated co-stimulatory molecule expression on cDCs after TLR2 signalling. In our hands, we have not been able to manipulate this response with exogenous IFNα in vitro or by placing IRF7-deficient cDCs within a type I IFN (and IRF7) sufficient host. Hence, our data support a model whereby IRF7 acts as a cell intrinsic regulator of cDCs activation independent of the availability of exogenous type I IFNs.

Tripartite motif-containing (TRIM) proteins comprise a family of type I IFN-inducible factors with diverse roles in the immune system [43]. Of particular relevance is TRIM30α, where bioinformatic analysis suggests the presence of a putative IRF7 binding sequence in its promoter region (Phillips and Kaye, unpublished). This protein acts to restrict NF-κB –mediated signalling downstream of TLR ligation by interacting with TAK1 and degrading TAB2 and TAB3 [44]. Interestingly TAB2 itself also contains a putative IRF7-binding element (Phillips and Kaye, unpublished), suggesting that IRF7 may be capable of regulating the activation of NF-κB directly and through the actions of TRIM30α. As enhanced costimulatory molecule expression is dependent on NF-κB [45], [46], it is possible that when this pathway is not correctly regulated, as may be the case in IRF7-deficient cDCs, that this results in a failure to regulate CD86 expression and thus leads to enhanced accumulation of this costimulatory molecule on cDCs. Further work will be required to confirm whether IRF7 does directly interact with TRIM30α, and to provide a molecular basis for our current observations.

The altered cytokine profile of cDCs lacking IRF7 and stimulated *in vitro* suggests an uncoupling of costimulatory molecule expression and cytokine production in response to TLR stimulation. In particular, the greatly reduced production of IL-12p70 by IRF7-deficient cDCs in response to TLR2 signalling was surprising, given the exaggerated expression of costimulatory molecules occurring simultaneously on these cells. This was unexpected, as previous reports have described exaggerated inflammatory cytokine production in the absence of IRF7, with MCMV infected B6.*Irf7* ^−/−^ mice having significantly elevated levels of serum IL-12p70 compared to wildtype animals [47]. Exacerbated pro-inflammatory cytokine production has also been reported in IRF7-deficient mice in a model of liver pathology, although this appears to be due to a failure in the IFNα-mediated induction of soluble IL-1Rα rather than any direct regulation of cytokine gene expression by IRF7 [41]. However, these studies did not address cytokine production from defined cell populations, and so the enhanced levels of systemic cytokines could be due to differential responses of distinct immune cell populations when deficient in IRF7. Of note, IRF1 and IRF8 cooperate to generate maximal IL-12 production [48], [49], [50], [51], [52], [53], [54], and IRF7 has been shown to interact with IRF8 [55] and is predicted to bind both IRF1 and IRF8 [23], [56]. Hence, IRF7-deficiency may affect IL-12 production via the direct and indirect activation of other IRFs.

Alongside their defective production of IL-12p70, cDCs deficient in IRF7 produced elevated levels of IL-10 in response to TLR ligation, sufficient to inhbit TH1 polarisation in vitro. This is similar to IRF1-deficient splenic DCs, and IRF5-deficient peritoneal macrophages [57] which show impaired IL-12 and enhanced IL-10 production *in vitro* [14]. However, splenic DCs from *Irf1^−/−^* mice have limited upregulation of costimulatory molecule expression in response to TLR ligation: a phenotype distinct from PAM_3_CSK_4_-stimulated splenic cDCs lacking IRF7 and indicating that differential mechanisms underlie these broadly similar observations.

In summary, this data reveals a novel role for IRF7 in the regulation of TLR2-induced costimulatory molecule expression by splenic cDCs *in vitro* and *in vivo*. This appears to be distinct from the regulation of pro-inflammatory cytokine production, as cDCs displaying exaggerated costimulatory molecule expression *in vitro* had a significantly impaired capacity for IL-12p70 production. In contrast IL-10 production was enhanced, indicating divergent effects of IRF7 on the regulation of cytokine production by cDCs. Although the molecular mechanisms remain to be experimentally determined, preliminary bioinformatic data suggest that interactions between IRF7 and regulators of TLR-induced NF-κB expression may underpin the altered expression of costimulatory molecules in IRF7-deficient cDCs.

## Materials and Methods

### Mice

C57BL/6 and B6J.CD45.1 mice were obtained from the Biological Services Facility (University of York) or supplied by Charles River Laboratories. B6.*Irf7^−/−^* mice were originally obtained from the RIKEN BioResource Centre (Ibaraki, Japan) with permission of T. Taniguchi, University of Tokyo. To generate microchimeric mice, B6J.CD45.1 mice were injected i.p with 20 mg/kg Busulfan (Pierre Fabre Pharmaceuticals, France) in 250 µl sterile 0.9% saline (Baxter, Norfolk, UK) and rested for 24 hours. Between 2×10^6^ and 8x10^6^ bone marrow cells from C57BL/6 or B6.*Irf7^−/−^* mice were transferred i.v. to Busulfan-treated mice and allowed to engraft over a period of 7–14 days before use. All animal care and experimental procedures were approved by the University of York Ethical Review Process and carried out under UK Home Office licence.

### Phenotypic Analysis of B6.*Irf7^−/−^* Mice by Flow Cytometry

C57BL/6 and B6.*Irf7^−/−^* mice were killed and spleens isolated. A single cell suspension was generated and red blood cells lysed. Splenocytes were washed and labelled on ice for 30 minutes with combinations of the following monoclonal antibodies; CD11c-PE-Cy7, MHCII-eFluor^450^, CD8α-FITC, CD4-APC, TLR2-PE, CD3ε-PE-Cy7, CD4-FITC, CD8α-PerCP, MHCII-eFluor^450^, CD19-PE, CD11b-eFluor^450^ and Gr-1-PE, all from eBioscience, and assessed by flow cytometry using a BeckmanCoulter CyAN and Summit software.

### Dendritic Cell Isolation

Splenic tissue was dissociated mechanically using a scalpel and digested in RPMI-1640 supplemented with 0.2mg/ml collagenase type IV/DNAse1 mix (Worthington Biochemical, NJ, USA) for 30 minutes at room temperature. A single cell suspension was generated and CD11c^+^ cells were enriched using a modified MACS magnetic separation protocol as previously described [58]. CD11c^hi^ or CD11c^hi^MHCII^hi^ cDCs were sorted to **∼**98–99% purity on a BeckmanCoulter MoFlo cell sorter.

### Activation of Splenic cDCs by TLR Agonists *in vitro*

CD11c^hi^ cDCs were cultured at 5×10^4^ cells/well in 50 µl complete RPMI for 24 hours. As indicated, DCs were stimulated 10 µg/ml PAM_3_CSK_4_ (Invivogen, San Diego, USA) in the presence/absence of 1000 U/ml IFNα (PBL Interferon Source, Piscataway, USA). Where indicated, *B6.Irf7^−/−^* splenic cDCs were cultured at a 1∶1 ratio with sorted congenic wildtype B6J.CD45.1 splenic cDCs. At indicated time points post-stimulation, cells were labelled with combinations of CD11c-PE, MHCII-efluor^450^, CD80-FITC and CD86-APC and assessed by flow cytometry for changes in surface expression of MHCII, CD80 and CD86, compared to unstimulated cDCs.

### cDC Cytokine Production

CD11c^hi^MHCII^hi^ cDCs were sorted from C57BL/6 and B6.*Irf7^−/−^* mice as described, plated in triplicate in complete RPMI at 1×10^6^ cells/ml and stimulated as indicated with 1 µg/ml LPS or 10 µg/ml PAM3CSK4 (Invivogen) for 24 hours, in the presence/absence of 1000 U/ml IFNα. Supernatants were assessed using a Quantikine ELISA (R&D Systems, Minneapolis, USA) for levels of IL-12p70 and IL-10.

### Dendritic Cell Activation by TLR Agonists *in vivo*

Microchimeric C57BL/6 **→** B6JCD45.1 and microchimeric B6.*Irf7^−/−^* **→** B6JCD45.1 mice were injected i.v. with 5 µg/mouse PAM_3_CSK_4_ (Invivogen, San Diego, USA) in 200 µl PBS or 200 µl PBS alone. After 24 hours, splenocytes isolated from individual mice were labelled with combinations of the following monoclonal antibodies; CD11c-PE, CD11c-FITC, CD45.1-PE-Cy7, MHCII-eFlour^450^, CD86-APC, all from eBioscience and CD80-FITC (BD Pharmingen) and analysed by flow cytometry for changes in costimulatory molecule expression.

### cDC and OTII.*Rag2^−/−^* T Cell Co-culture

5×10^4^ cDCs were cultured with 1×10^5^ CFSE (3 µM)- labelled OTII T cells for 5 days in the presence of 5 nM of the OTII CD4^+^T cell epitope, OVA_(323–339)_. After 7 days of culture, cells were restimulated with PMA & Ionomycin and cytokine production assessed by intracellular cytokine staining and flow cytometric analysis. Briefly, cells were restimulated for 90 minutes at 37°C, 5% CO_2_ with 10 ng/ml PMA and 1 **µ**g/ml Ionomycin before the addition of 1 **µ**g/ml Brefeldin A (all from Sigma-Aldrich, UK) for the final 4.5 hours of culture. Cells were washed and labelled for 30 minutes on ice with the following monoclonal antibodies: TCRβ-APC (H57–957- eBioscience) and CD4-PerCP (RM4–5- BD Pharmingen). Cells were fixed, washed and labeled intracellulary for 45 minutes on ice with IFNγ-eFluor^450^ (XMG1.2) in PBS containing 1% saponin and 1% BSA. After staining, cells were washed and data acquired on a CyAN-ADP flow cytometer (Beckman Coulter, USA). Subsequent analysis was performed using FlowJo software (Treestar, USA).

### Statistical Analysis

Statistical analysis was performed using a student’s t test where p<0.05 was considered significant.

## Supporting Information

Figure S1 **IRF7 deficiency does not affect spleen cell composition.**The frequency of **A**, splenic CD3ε^+^ T cells, **B**, CD4^+^ and CD8α^+^ T cells, **C**, CD19^+^MHCII^+^ B cells and **D**, CD11b^+^ monocytes and CD11b^+^Gr-1^+^ neutrophils were assessed by flow cytometry in steady state C57BL/6 (open bars) and B6.*Irf7* ^−/−^ (closed bars) mice. In A., dot plot shows ungated spleen cell samples stained with viability eFluor-780. The high number of dead lymphocytes (upper population) is a consequence of the collagensae treatement used to extract cDCs from tissue. In all subsequent panels, dead cells have been gated out. Data show representative flow plots and where quantified show the mean frequency ± SEM of indicated cell type in spleens of 4–5 mice per group. Representative of three separate experiments.
(TIF)

Figure S2 **Limited impact of IRF7 deficiency on cDC responses to TLR3, TLR4 and TLR9 triggering** ***in vitro*** **.** Splenic CD11c^hi^ cDCs were sorted from C57BL/6 and B6.*Irf7^−/−^* mice. Cells were stimulated with 100 µg/ml Poly (I:C), 1 µg/ml LPS or 10 µg/ml ODN:1668. At the indicated times post-stimulation, cells were removed and assessed by flow cytometry. **A**–**I**, fold increases in surface expression of CD80, CD86 and MHCII on cDCs at the indicated time point after stimulation with Poly I:C (A–C), LPS (D–F) and ODN:1668 (G–I), over unstimulated cDCs **A**–**I** show mean fold increase ± SEM in surface expression of indicated proteins on cDCs from C57BL/6 (open bars) or B6.*Irf7* ^−/−^(closed bars) mice, compared to unstimulated cDCs from the same strain. Data are pooled from three individual experiments. * = p<0.05 ** = p<0.01, *** = p<0.001.
(TIF)

Figure S3 **Heterogeneous hyperactivity in IRF7-deficient cDCs** ***in vitro*** **.** Fold changes in MFI of indicated surface markers on CD11c^hi^ cDCs as in **Fig.** **2** gated on MHCII^hi^CD86^hi^ cells (**A**). **B**–**D** show mean fold increase ± SEM in surface expression of indicated proteins on cDCs from C57BL/6 (open bars) or B6.*Irf7* ^−/−^(closed bars) mice, after PAM_3_CSK_4_ stimulation compared to unstimulated cDCs from the same strain, **E** shows the mean frequency ± SEM of MHCII^hi^CD86^hi^ cDCs of each genotype at the indicated time point after stimulation. Data are pooled from three individual experiments. * = p<0.05 ** = p<0.01, *** = p<0.001.
(TIF)
