# Supplementary figures and images for: IRF7 Regulates TLR2-Mediated Activation of Splenic CD11chi Dendritic Cells
Source: PLoS One. 2012 Jul 16;7(7):e41050. doi: 10.1371/journal.pone.0041050 (PMC3398003; doi:10.1371/journal.pone.0041050)

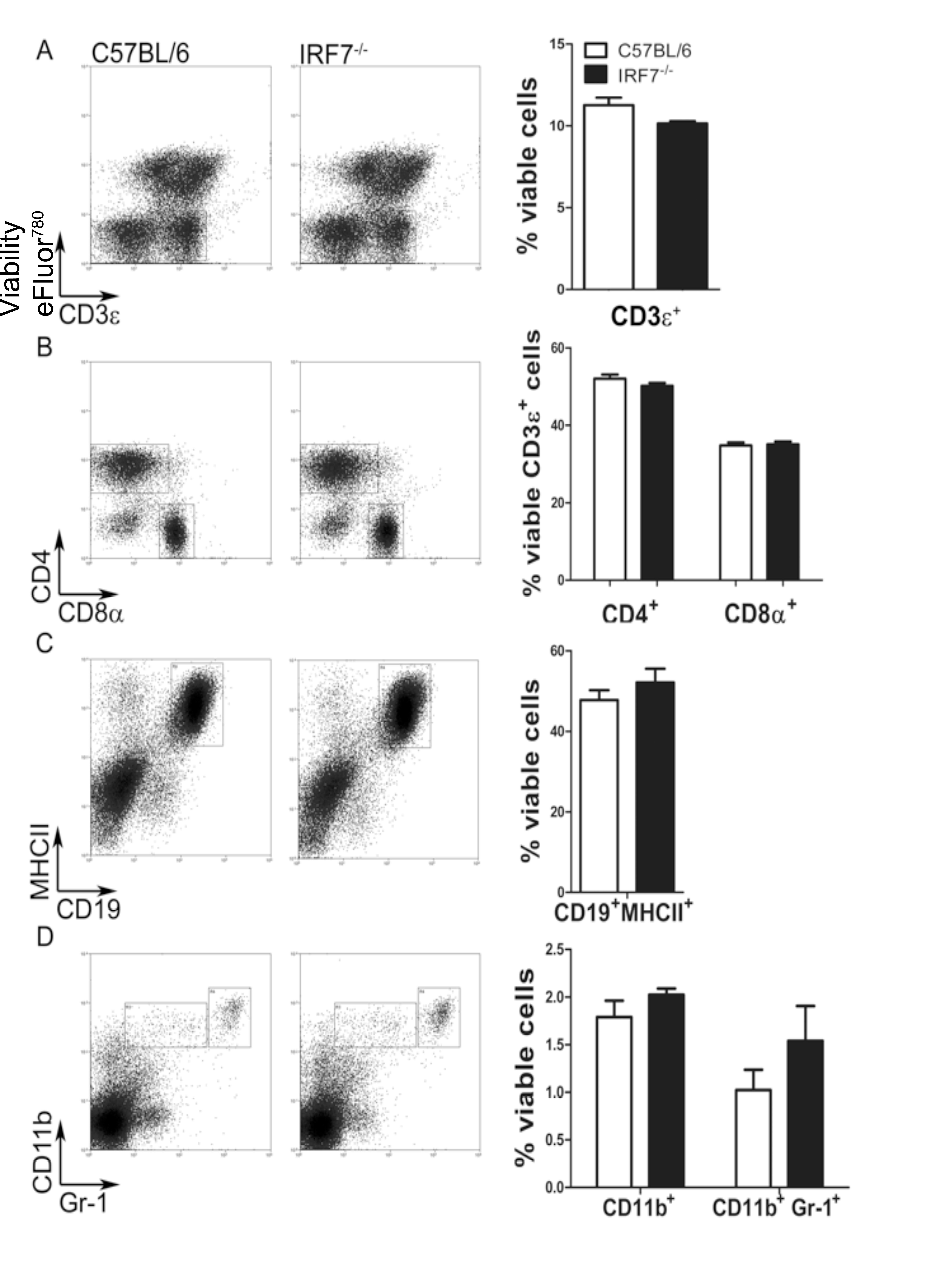

Supplement: Figure S1 — IRF7 deficiency does not affect spleen cell composition.The frequency of A, splenic CD3ε+ T cells, B, CD4+ and CD8α+ T cells, C, CD19+MHCII+ B cells and D, CD11b+ monocytes and CD11b+Gr-1+ neutrophils were assessed by flow cytometry in steady state C57BL/6 (open bars) and B6.Irf7 −/− (closed bars) mice. In A., dot plot shows ungated spleen cell samples stained with viability eFluor-780. The high number of dead lymphocytes (upper population) is a consequence of the collagensae treatement used to extract cDCs from tissue. In all subsequent panels, dead cells have been gated out. Data show representative flow plots and where quantified show the mean frequency ± SEM of indicated cell type in spleens of 4–5 mice per group. Representative of three separate experiments. (TIF) [file pone.0041050.s001.tif]

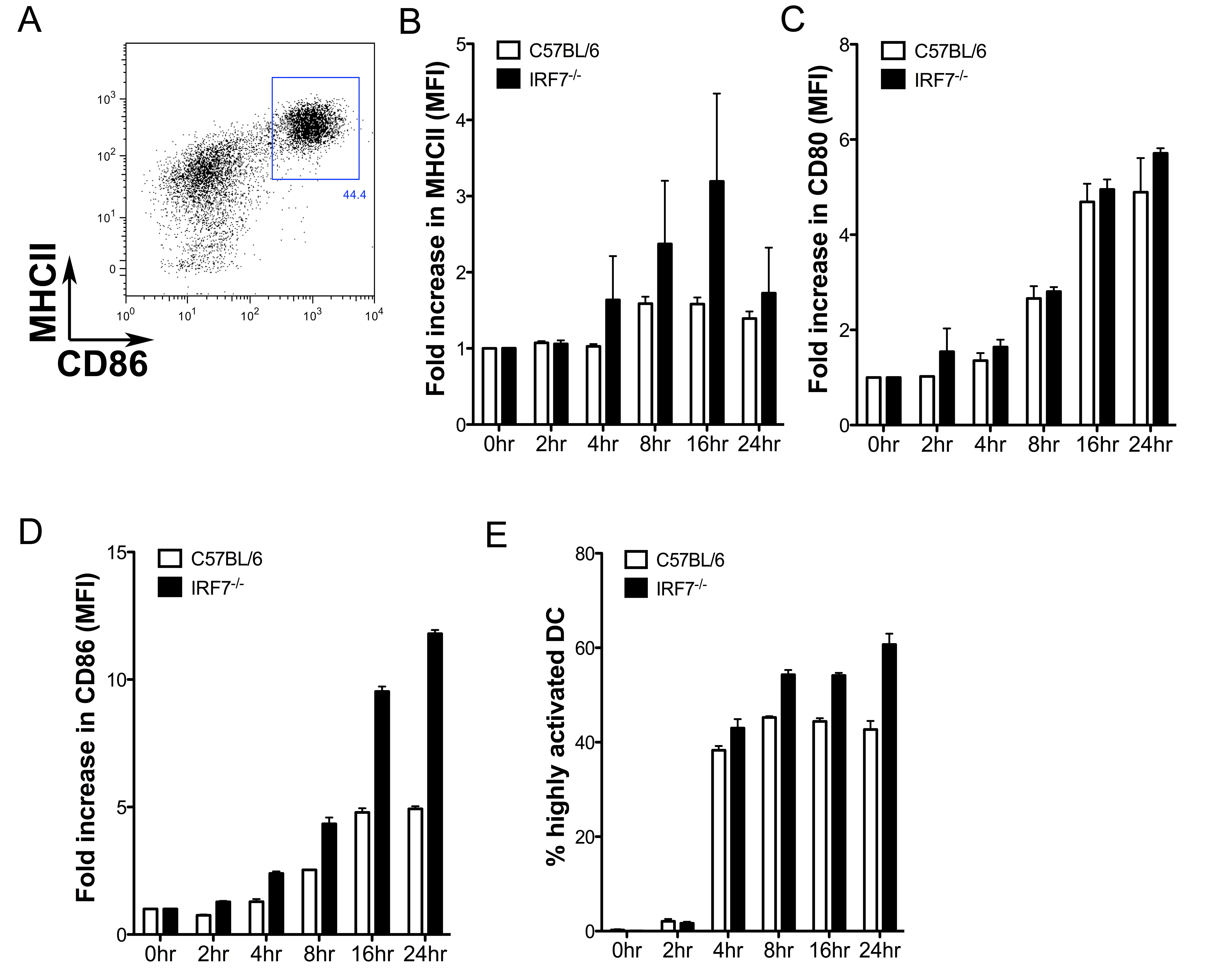

Supplement: Figure S3 — Heterogeneous hyperactivity in IRF7-deficient cDCs in vitro . Fold changes in MFI of indicated surface markers on CD11chi cDCs as in Fig. 2 gated on MHCIIhiCD86hi cells (A). B–D show mean fold increase ± SEM in surface expression of indicated proteins on cDCs from C57BL/6 (open bars) or B6.Irf7 −/−(closed bars) mice, after PAM3CSK4 stimulation compared to unstimulated cDCs from the same strain, E shows the mean frequency ± SEM of MHCIIhiCD86hi cDCs of each genotype at the indicated time point after stimulation. Data are pooled from three individual experiments. * = p<0.05 ** = p<0.01, *** = p<0.001. (TIF) [file pone.0041050.s003.tif]
